# Single port/incision laparoscopic surgery compared with standard three-port laparoscopic surgery for appendicectomy - a randomised controlled trial

**DOI:** 10.1186/1745-6215-13-201

**Published:** 2012-10-30

**Authors:** Momin Malik, Kirsty McCormack, Zygmunt H Krukowski, Alison McDonald, Gladys McPherson, Jonathan A Cook, Irfan Ahmed

**Affiliations:** 1 Aberdeen Royal Infirmary, Foresterhill, Aberdeen, AB25 2ZN, UK; 2 Health Services Research Unit, Health Sciences Building, Foresterhill, University of Aberdeen, Aberdeen, AB25 2ZD, UK

**Keywords:** Appendicitis, Appendicectomy, Laparoscopic, Single port, Single incision, SCARLESS

## Abstract

**Background:**

Laparoscopic surgery has become the preferred approach for many procedures because of reduced post-operative pain, better recovery, shorter hospital stay and improved cosmesis. Single incision laparoscopic surgery is one of the many recent variants where either standard ports or a specially designed single multi-channel port is introduced through a single skin incision. While the cosmetic advantage of this is obvious, the evidence base for claims of reduced morbidity and better post-operative recovery is weak. This study aims to compare the effectiveness of single port/incision laparoscopic appendicectomy with standard three-port laparoscopic appendicectomy in adult patients at six weeks post-surgery. We also wish to assess the feasibility of a multicentre randomised controlled trial comparing single port/incision laparoscopic surgery with standard three-port laparoscopic surgery for other surgical techniques.

**Methods and design:**

Patients diagnosed with suspected appendicitis and requiring surgical treatment will be randomised to receive either standard three-port or single incision laparoscopic surgery. Data will be collected from clinical notes, operation notes and patient reported questionnaires. The following outcomes will be considered:

1. Effectiveness of the surgical procedure in terms of:

•patient reported outcomes

•clinical outcomes

•resource use

2. Feasibility of conducting a randomised controlled trial (RCT) in the emergency surgical setting by quantifying:

•patient eligibility

•randomisation acceptability

•feasibility of blinding participants to the intervention received

•completion rates of case report forms and patient reported questionnaires

**Trial registration:**

ISRCTN66443895 (assigned 10 March 2011, first patient randomised 09 January 2011)

## Background

Appendicectomy is one of the most commonly performed surgical procedures in general surgery. In England (2010 to 2011), appendicectomy resulted in 47,145 operations; 41,458 of which were for an emergency admission [1]. Laparoscopic surgery is the preferred approach for many abdominal procedures because of reduced postoperative pain, more rapid recovery and improved cosmesis, which follow a successful operation compared with a conventional single large incision. Whilst the long term clinical result may be similar [2], the perception amongst many patients and surgeons of advantage in terms of these short-term outcomes is a powerful influence on practice. There are continuing developments to laparoscopic surgery to reduce the size, number and placement of incisions to both improve the cosmetic appearance and reduce abdominal wall trauma.

One of the recent innovations is Single Incision Laparoscopic Surgery. This can be either insertion of multiple ports through a small incision or through a proprietary device with multiple channels. The fundamental difference to conventional multi-port laparoscopic surgery is to place all the ports through a single incision which, when sited in the umbilicus, can result in no visible scar in the abdominal wall.

The current literature largely comprises case reports and small series detailing single port methods [3-9]. The technique has been used to perform a large variety of procedures including appendicectomy, cholecystectomy, nephrectomy, hysterectomy, oophorectomy, adrenalectomy, gastric bypass, Nissen fundoplication, hernia repair, splenectomy, colon resection and liver resection. Apart from a handful of reported randomised controlled trials (RCTs) [3-9] the evidence base is weak and insufficient to inform practice and robustly assess claims of reduced pain and morbidity with improved cosmesis and faster recovery are unsubstantiated [10-15]. In general, it is perceived that the single port/incision technique takes longer initially than conventional laparoscopic surgery and the differences in costs and safety are unknown.

Nevertheless, there is considerable interest in introducing single port/incision surgery and there are a large number of training courses. The public perception is that it might become the procedure of choice if it becomes available [16]. It is crucial that the technique be critically evaluated during the introductory phase of implementation to provide objective data to inform further adoption and evaluation. However, the difficulty of undertaking such an evaluation has been succinctly stated in Buxton’s law: “It is always too early [for rigorous evaluation] until, unfortunately, it’s suddenly too late” [17]. The introduction of laparoscopic surgery for Cholystectomy is a vivid example of the *ad hoc* nature in which a new surgical intervention can be introduced into practice and the difficulties of conducting rigorous evaluations of its value [18]. Ideally, a definitive evaluation requires a large, multicentre RCT of single port versus three-port surgery. Currently, there is a paucity of data to help plan and design such a large RCT. Additionally, further refinement of the single port/incision methodology is needed. There is, therefore, an urgent need for a well conducted feasibility study to provide preliminary results and inform the planning of such a large RCT (for example, how to define the intervention, which outcomes to use and when; likely throughput rates; estimates of conversion rates). It is hoped that the results of this study will lead to a large multicentre RCT of single versus standard three-port laparoscopic surgery.

This study will compare the effectiveness of single port/incision laparoscopic appendicectomy with standard three-port laparoscopic appendicectomy in adult patients at six weeks post-surgery. Additionally, it will inform the feasibility of more complex single port techniques, such as cholecystectomy. Appendicectomy is the focus of this study because it is a common and relatively simple procedure to undertake. Currently, no RCTs of single port versus three-port surgery have been published [8].

The specific objectives are:

•To compare the interventions in terms of patient reported outcomes, clinical measures and resource use.

•To assess the feasibility of a randomised controlled trial evaluating another single port/incision operation by quantifying patient eligibility and acceptability, feasibility of blinding participants to the intervention received and surgeon perception of interventions.

## Methods and design

**Figure 1:**
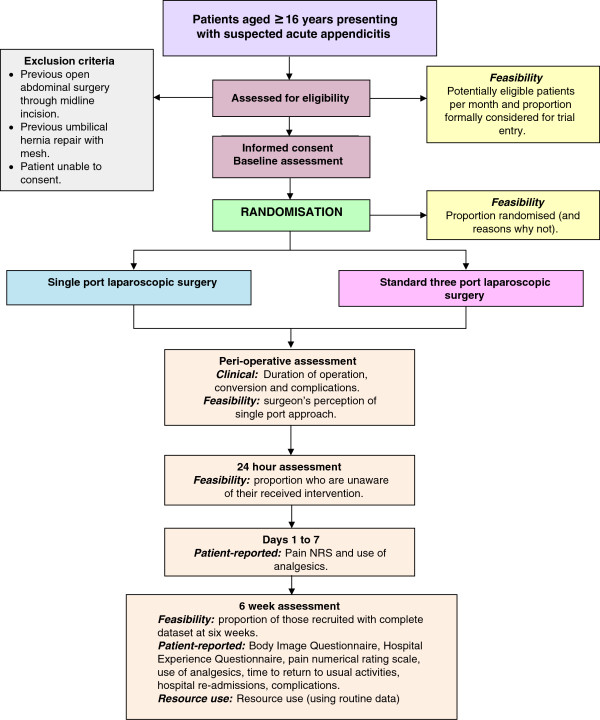
Trial flow diagram.

Figure 1 provides an overview of the trial’s methodology.

### Inclusion and exclusion criteria

#### Inclusion criteria

Patients aged 16 years and over presenting with suspected appendicitis for whom laparoscopic surgical management is judged appropriate are eligible for inclusion.

#### Exclusion criteria

1. Patients who have had previous open abdominal surgery through midline incision.

2. Patients who have had previous umbilical hernia repair with mesh.

3. Patients unable to consent.

### Trial interventions

Participants will receive the allocated intervention, either single port/incision laparoscopic surgery (SPILS) or standard three-port laparoscopic appendicectomy surgery. The surgical interventions will be delivered or supervised by a surgeon who has expertise in the specific intervention. All participating surgeons will have completed appropriate surgical training. Further details of the interventions are given below:

#### SPILS

A single intra-umbilical incision will be made and a multi-channel port will be inserted. A 5 mm, 30 degree telescope will be used to visualise the operative field. Conventional laparoscopic instruments will be used for the procedure. Roticulating/curved instruments will be available and used if required. Use of any additional instruments or ports will be recorded. The musculo-aponeurotic layers of the port site will be closed with absorbable sutures before closing the skin incision.

#### Standard three-port laparoscopic surgery

Pneumoperitoneum will be established by an open technique through an intra/supraumbilical incision with a 10 to 12 mm port for initial pneumoperitoneum and inspection. A further 5 or 10 mm port will be used in the left iliac fossa (depending on the availability of 5 mm laparoscopes) and a 5 mm port will be used in the hypogastrium. Standard laparoscopic instruments will be used for the procedure as per existing hospital protocol. The musculo-aponeurotic layers of port sites of 10 mm and over will be closed with absorbable sutures before closing the skin.

The routine surgical technique will be dissection of the mesoappendix from the appendix with diathermy and division of the appendix base between two endoloops. In more complicated cases, alternative techniques may be used. Any variations to the regimen with justification will be recorded.

A standard pain relief policy will be followed, where possible. This will include one or more of the following postoperative analgesics: paracetamol (1g QID); dihydrocodeine, diclofenac and morphine (doses will be titrated and recorded).

### Identification and enrolment of potential participants

Patients likely to require surgery for acute appendicitis and who meet the eligibility criteria will be identified in the general surgery units by the consultant or designated team member. The consultant or team member will introduce the study to the patients and provide further details of the study by means of the Patient Information Sheet.

The participants will keep a copy of the Patient Information Sheet and the consent form (one copy will be filed in the hospital notes, one given to the participants and the top copy returned to the SCARLESS co-ordinating office in Aberdeen). A letter and General Practitioner (GP) Information Sheet will be sent to the participant’s GP. Participants who initially agree to enter the study but later decide to withdraw or become unable to continue will be asked for consent to enable us to access relevant NHS data in the future.

Patients who are ineligible or who do not agree to participate in the study will be logged anonymously along with a minimum dataset including gender, year of birth and reason(s) for declining (if offered).

### Randomisation and allocation

Participants will be recruited from a single centre, Aberdeen Royal Infirmary (UK). Following consent and collection of baseline data, the local consultant/designated team member will randomise the patient.

Participants will be randomised to one of the two study groups in equal proportion using the randomisation application at the trial office at the Centre for Healthcare and Randomised Trials (CHaRT), Aberdeen. Randomisation will be stratified by gender and computer generated permuted blocks of varying size used within each stratum. This randomisation application will be available 24 hours a day, seven days a week as an Interactive Voice Response (IVR) telephone system. Due to the acute nature of the admission to surgery and potential difficulty in tracking patients, date of birth will also be recorded in the randomisation process and available, if necessary, for use in addition to the study number to identify patients. The date of birth will not be openly available.

### Concealment of group allocation to participants

The feasibility of concealing group allocation from the participants and the ward staff will be investigated. Although blinding in the theatre is not possible given this is a surgical trial, participants will not be informed after their surgery of the procedure actually carried out. Blinding of participants will be attempted by using three hypoallergenic dressings applied at the umbilicus, in the left lower quadrant and hypogastrium unless conversion to open abdomen is required.

### Sample size

As there were no published RCTs comparing single port laparoscopic appendicectomy with standard three-port laparoscopic appendicectomy when the study was designed [8], a formal sample size calculation based upon previous data was not possible. A sample of 80 participants recruited is anticipated over the seven-month recruitment phase. Adopting a 5% two-sided significance level, this would allow an effect size (Cohen’s d) of 0.65 to be detected with 80% power for patient reported measures, such as the Body Image Questionnaire [19]. Binary feasibility measures are likely to be estimated with a (one-group) confidence interval of between 10 and 20% depending upon the corresponding event rate [19].

### Subsequent tasks

Following formal trial entry -

The Study Office will:

i. Inform the participant’s GP (by letter enclosing information about SCARLESS and Study Office contact details).

ii. Process the pain Numerical Rating Scale (NRS) seven-day diary and the six-week participant questionnaire.

iii. Collect the feasibility measures.

The consultant or team member will:

i. Provide the original signed consent form to the Study Office and file the Hospital Copy of the form in the hospital notes along with information about SCARLESS.

ii. Inform the ward and theatre staff as appropriate of the participant’s study participation and intervention allocation.

iii. Complete case report forms (CRFs) as appropriate and either enter the data directly onto the SCARLESS website or forward the hard copies of the CRFs to the co-ordinating office at CHaRT for electronic data entry. The data to be collected include data required to complete randomisation; intra- and postoperative.

iv. Return all study documentation to the Study Office (CHaRT, Aberdeen) for archive.

### Monitoring the participants

Participants will be contacted by post and phone as appropriate. In case of non-return of questionnaires, the participant will be sent a postal reminder or receive a telephone call.

### Data collection and processing

Follow-up will continue for six weeks from the date of operation. Clinical data will be collected on participants who will need to be followed-up in clinic, as part of their treatment plan.

Participants will be assessed pre-operatively to confirm eligibility and peri-operative data collected. Patient reported outcomes (PROs) will be collected by diary completed on days one to seven following surgery and by postal questionnaires at six weeks post-surgery. The components of participant follow-up are shown in the Table 1.

**Table 1:** Source and timing of measures

**Outcome measures**	**Source**	**Timing**		
		**Peri- operative**	**Days one to seven diary**	**Six weeks**
BMI, ASA grade	Case Report Form (CRF)	▲		
Duration of operation, intra-operative complication rates, conversion rates, surgeon’s perception of single port approach	CRF	▲		
Intervention received	CRF	▲		
Body Image Questionnaire (BIQ) [20]	Participant Questionnaire (PQ)			▲
Hospital Experience Questionnaire (HEQ) [20]	PQ			▲
Pain Numerical Rating Scale (NRS)	PQ		▲	▲
Use of analgesics	CRF and PQ	▲	▲	▲
Time to return to usual activities	PQ			▲
Hospital re-admissions	CRF and PQ			▲
Complications (for example, infection, port-site hernia)	CRF and PQ	▲		▲

### Outcome measures

#### Primary effectiveness outcomes

The patient reported outcome measure is the patient reported cosmesis and body image using the Body Image Questionnaire (BIQ) [20] at six weeks; participants will be asked five questions about their body image using the scale on a 4-point Likert scale of:’no, not at all’; ‘a little bit’; ‘quite a bit’; ‘yes, extremely’, two questions about their incisional scar to be rated on a scale of 1 (very unsatisfied/revolting) to 7 (very satisfied/beautiful), one further question regarding the scar using a 10-point Numerical Rating Scale (NRS), and a question regarding confidence on a 10-point numerical scale from 1 (not very confident) to 10 (very confident). The clinical outcome of severity of pain (Pain NRS) will be measured using a pain scale (scale from 0 (no pain) to 10 (worst imaginable pain)) at one to seven days.

#### Other patient-reported outcomes

Patient reported measures are the Hospital Experience Questionnaire (HEQ) [20] at six weeks where participants will be asked four questions about their experience in hospital (prior to the operation, treatment received, pain after operation and time to normal eating) to be rated subjectively using either a 4- or 5-level Likert scale (‘much too long’ to ‘much too short’; ‘very bad’ to ‘very good’; ‘no pain at all’ to ‘a lot of pain’; ‘no, not at all’ to ‘I cannot remember’) and one rating question on their view of the importance of different items (hospital stay, size of scar, no complications, pain after surgery, and resuming normal activities and diet). Additionally, any analgesic usage and time to return to normal activities will be collected.

#### Clinical outcomes

Analgesic use; duration of operation (minutes) and complication rates; conversion rates; infection rates (intra-abdominal and wound); related hospital re-admission rates up to six weeks; reoperation rates and port-site hernia up to six weeks.

#### Feasibility measures

Feasibility measures include eligible patients per month; proportion formally considered for trial entry; proportion randomised (and reasons why not); proportion who are unaware of their received intervention at 24 hours; proportion of those recruited with complete data set at six weeks; surgeon’s perception of SPILS approach and the suitability of available equipment.

#### Resource use

Resource use will be limited to duration of operative procedure, theatre time and use of disposable instruments.

### Statistical analyses

For feasibility measures, such as the proportion of eligible patients who consent to randomisation, the frequency and corresponding 95% confidence interval will be calculated. Patient-reported and clinical measures will be summarised using appropriate summary measures (for example, frequency or mean and standard deviation) for each treatment group. The treatment groups will be compared at the two-sided 5% significance level. BIQ and pain NRS (area under the curve over a seven-day period) will be analysed using an independent *t*-test. Other outcomes will be assessed using standard statistical methods as appropriate, for example, comparison of proportions Newcombe’s CI method [21] or chi-squared test for trend [22], and independent *t*-test for binary and continuous outcomes respectively. Corresponding 95% confidence intervals will also be calculated. A single principal analysis is anticipated at the end of the study following intention to treat principle (grouped according to allocation). No imputation for missing data will be carried out. All analyses will be conducted using Stata 12 [23].

### Timetable of work

The planned study duration is 12 months. The main milestones are: months 1 to 2 NHS approvals; months 3 to 9 patient recruitment; months 10 to 11 complete participant follow up at 6 weeks; months 11 to 12 analysis of data, interpretation of results and report writing.

**Figure 2:**
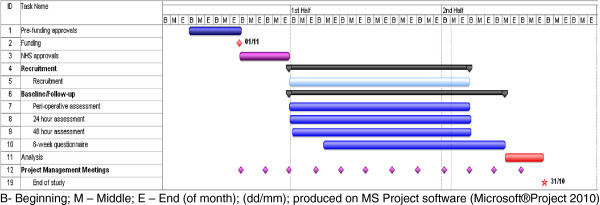
SCARLESS Gannt Chart.

The timetable is described in the Gantt chart in Figure 2.

## Endnotes

### Data protection

The trial will comply with the Data Protection Act 1998 [24] and regular checks and monitoring will be in place to ensure compliance. Data will be stored securely in accordance with the Act and archived to a secure data storage facility. The consent form will state that other researchers may wish to access (anonymised) data in the future. CHaRT’s senior IT Manager (in collaboration with the Chief Investigator) will manage access rights to the data set.

### Sponsorship

The study is co-sponsored by the University of Aberdeen and NHS Grampian.

### Retention of data

It is intended that data will be retained for at least five years following the end of the study.

### Safety concerns

The SCARLESS trial involves laparoscopic surgical operations for appendicectomy. Possible complications and consequences are:

#### Anaesthesia

All operations carried out under general anaesthesia carry a risk of death, muscle paralysis, technical problems, adverse drug reactions and allergic responses.

#### Abdominal surgery

All abdominal operations carry a risk of death, morbidity, including wound infection, wound hernias, damage to abdominal viscera, bleeding intra and post-operatively, bowel obstruction, respiratory infections, lung collapse, thrombo-embolic complications, complications secondary to existing co-morbidity, for example, ischaemic heart disease and diabetes.

#### Laparoscopic abdominal surgery

Specific complications related to the laparoscopic approach include inadvertent injury to abdominal viscera due to the restricted view associated with laparoscopy, failure to appreciate the extent of pathology, possible sub-optimal repair of intra-operative injuries, inadequate closure of port sites with resulting early and late herniation, which may result in bowel obstruction. With the single port/incision laparoscopic surgery (SPILS) the loss of triangulation of instrumentation, generally considered necessary in laparoscopic surgery, limits surgeon’s access and manoeuvrability.

### Procedure for reporting untoward and related serious adverse events (SAEs)

The UK NHS National Research Ethics Service guidelines for reporting serious adverse events will be followed [25]. For the purpose of SCARLESS, all SAEs, defined as an event resulting from a participant’s appendicectomy treatment that is life threatening, requires prolongation of an existing hospital admission or readmission, results in significant incapacity/disability or is considered to be an important medical event by the clinical team, will be recorded on the Serious Adverse Event Report form. In addition, SAE forms will record all deaths for any cause during the course of the study.

A SAE that is both:

•related (resulted from administration of any of the research procedures) and

•unexpected (that is, the type of event that is not listed as a possible expected complication)

will be notified to the appropriate authorities (Research Ethics Committee (REC) and co-sponsors) within the timelines outlined in the guidelines, as detailed below.

### Reporting responsibilities of the CI

The Chief Investigator (CI) or deputy will be automatically notified of all SAEs. If, in the opinion of the local surgeon and the CI, the event is confirmed as being related and unexpected, the CI will submit a report to the main REC and the study sponsors within 15 days of the CI becoming aware of it.

As the trial arm to which participants are allocated cannot be blind to the operating surgeons or theatre staff after randomisation has occurred, unblinding is not an issue in this trial. A record of the operative procedures actually carried out will be available in the medical notes if required clinically.

### Ethical issues and arrangements

The North of Scotland Ethics Committee (NOSRES) reviewed and approved this study on 08 December 2010 (REC reference number: 10/S0802/77).

### Risks and benefits

The benefit to the participants participating in the trial is the chance of receiving a less invasive treatment for their appendicectomy. We believe this study does not pose any specific risks to individual participants beyond those of any laparoscopic surgery, provided sound clinical judgement is exercised to convert to multi-port or conventional surgery when necessary.

### Information about risks and benefits and informed consent

Participants are informed of known risks by means of the Patient Information Sheet. Patients will have surgery whether they are in the study or not and we anticipate no additional risk to those who are randomised. Patients who are ineligible or decline participation in the study will have standard open or three-port laparoscopic surgery as these are the standard types of surgeries undertaken at Aberdeen Royal Infirmary. SPILS will only be available to patients randomised within the trial.

## Abbreviations

ASA: American Society of Anaesthiologists; ARI: Aberdeen Royal Infirmary; BIQ: Body Image Questionnaire; BMI: Body Mass Index; CHaRT: Centre for Healthcare Randomised Trials; CI: Chief Investigator; CSO: Chief Scientist Office; CRF: Case Report Form; GP: General Practitioner; GCP: Good Clinical PracticeHEQ Hospital Experience Questionnaire; ISRCTN: International Standard Randomised Controlled Trial Number; IT: Information Technology; IVR: Interactive Voice Response; mm: Millimetres; MRC: Medical Research Council UK; NHS: National Health Service; NOSRES: The North of Scotland Research Ethics Committee; NRS: Numerical Rating Scale; PMG: Project Management Group; PQ: Participant Questionnaire; PROs: Patient Reported Outcomes; QID: 4 Times a day; REC: Research Ethics Committee; RCT(s): Randomised Controlled Trial(s); SAEs: Serious Adverse Events; SCARLESS: Single Centre Appendicectomy RCT: Laparoscopic vs Endoscopic Single-port Surgery; SPILS: Single Port/Incision Laparoscopic Surgery using either a proprietary device or multiple trocars inserted through a single incision; TSC: Trial Steering Committee; UK: United Kingdom; vs: Versus.

## Competing interests

The authors’ declare that they have no competing interests.

## Authors’ contributions

IA (Chief Investigator), KM, ZHK and JAC are grant holders and designed the study. AM and GM developed the protocol, website, standard operating procedures, data collection and monitoring. MM contributed to protocol development and data collection, and was responsible for coordinating and implementing the surgical protocols and editing of the trial protocol for publication. All authors read and approved the final manuscript.

## Authors’ information

Aberdeen Royal Infirmary is ideally suited to perform this feasibility study. It is a large teaching hospital with a high through-put of emergency patients admitted for appendicectomy. The grant holders have complementary skills and experience. Irfan Ahmed is a consultant general surgeon with extensive experience in laparoscopic surgery and is pioneering techniques in SPILS. He is a recognised national trainer in single-port techniques. Prof. ZHK has extensive experience in laparoscopic surgery and a track record in research and clinical trials. MM is a clinical fellow in hepatobiliary and advanced laparoscopic surgery. JAC is a methodologist with extensive experience in all aspects of clinical trial design, conduct and analysis with particular expertise in the evaluation of surgical interventions. KM is an experienced health services researcher with a wide breadth of experience in clinical trials and a particular interest in laparoscopic surgery. GM is an experienced trialist with several years' experience of programming and data management in clinical trials. AM is an experienced trialist with extensive experience in trial management.
